# The relationship between fatigue levels and psychosocial adjustment in elderly individuals with chronic obstructive pulmonary disease: A descriptive study

**DOI:** 10.1007/s11845-025-03882-y

**Published:** 2025-01-28

**Authors:** Ebru Akbaş, Sümeyra Buse Filikci

**Affiliations:** 1 Faculty of Health Sciences, Department of Gerontology, Mehmet Akif Ersoy University, Burdur, Turkey; 2 Nursing Department, School of Susehri Health High, Cumhuriyet University, Sivas, Turkey

**Keywords:** Chronic disease, Chronic obstructive pulmonary disease, Fatigue, Psychosocial adjustment

## Abstract

**Background:**

Chronic Obstructive Pulmonary Disease (COPD) is associated with physical limitations and significant social, psychological, and behavioral challenges. This study investigates the relationship between fatigue levels and psychosocial adjustment in COPD patients, considering their sociodemographic characteristics.

**Methods:**

A descriptive study was conducted with 160 COPD patients hospitalized in the Pulmonology Department of a university hospital. Data were collected via face-to-face interviews using a patient information form, the COPD and Asthma Fatigue Scale (CAFS), and the Psychosocial Adjustment to Illness Scale-Self-Report (PAIS-SR). Statistical analyses included Independent Sample *t*-test, One-Way ANOVA, Mann–Whitney *U* test, Kruskal–Wallis test, and Spearman correlation analysis, with significance set at *p* < 0.05.

**Results:**

The mean age of participants was 68.70 ± 9.41 years; 71.9% were male, and 67.9% had COPD for over six years. Most participants (74.4%) reported limitations in daily living activities due to the disease, and 91.9% reported having social support. Mean scores were 58.03 ± 15.80 on the CAFS and 64.19 ± 6.41 on the PAIS-SR. Significant differences were observed in fatigue and psychosocial adjustment scores based on gender, social support, and disease impact on daily activities (*p* < 0.05). A weak positive correlation was found between fatigue levels and psychosocial adjustment (*p* < 0.05).

**Conclusions:**

COPD patients experience moderate-to-high fatigue levels and challenges in psychosocial adjustment, with fatigue negatively influencing adjustment. Interventions should focus on enhancing coping strategies, addressing psychosocial needs, and leveraging social support systems to improve patient outcomes.

## Introduction

With increasing life expectancy and an aging population, the number of people affected by chronic diseases is rising. Chronic diseases are characterized by their slow impact on an individual’s health, long-term nature, recurrence, and incurability, necessitating effective management strategies [1]. Cardiovascular and respiratory diseases, diabetes, and cancer are examples of major chronic diseases that are highly prevalent worldwide [2, 3]. These prolonged conditions can significantly impact all aspects of an individual's life [2, 4], often leading to loss of physical strength, pain, distress, depression, reduced quality of life, and even job loss [5, 6]. Additionally, chronic diseases can evoke feelings such as deteriorated body image, inability to fulfill personal roles, and dependence on others [4]. This, in turn, creates a burden on healthcare resources and leads to increased mortality [7, 8]. Chronic obstructive pulmonary disease (COPD) is one such condition that carries a high disease burden and is projected by the WHO to become the third leading cause of death by 2030 [9].

Chronic Obstructive Pulmonary Disease (COPD) is one of the most common preventable and treatable respiratory diseases, characterized by airflow restriction that results from significant, long-term exposure of the respiratory tract to harmful substances or gases [10, 11]. COPD is marked by persistent airflow limitation and severe shortness of breath [12]. The second most prevalent symptom after dyspnea is fatigue [13]. Although fatigue is a common, general symptom, it is among the least recognized and under-treated symptoms [14]. Fatigue can be experienced in two distinct ways. In healthy individuals, fatigue is typically short-lived and can be alleviated with rest throughout the day. However, for patients with chronic diseases, fatigue is continuous and can lead to burnout, affecting their ability to rest and nourish themselves adequately [13].

Fatigue can have negative consequences on health, as it limits patients’ activities of daily living, leads to a poor prognosis, and may be an indicator of mortality [15, 16]. Stridsman et al. [15] reported a correlation between fatigue and health status in COPD patients, indicating that fatigue can reduce both the physical and psychological well-being of these individuals. A qualitative study by Kouijzer et al. [17] also found that patients’ fatigue significantly impacts their daily lives, underscoring its role in reducing their quality of life. Furthermore, Yang et al. [11] emphasize that fatigue has a strong negative impact on the quality of life, physical health, and mental health of COPD patients, suggesting that healthcare professionals should prioritize screening, counseling, and health education on fatigue, particularly for patients with moderate to severe COPD.

In this context, individuals with chronic diseases not only struggle with the symptoms of the disease itself but also face challenges in maintaining their daily lives amidst the complications caused by the disease and its treatments [4]. Due to limitations in their activities of daily living, these patients often find it challenging to maintain their lives in the same way as others.

Having a chronic illness not only leads to physical limitations but can also restrict an individual’s social life, education, professional opportunities, and activities such as finding a job. Therefore, when examining chronic diseases as a long-term burden, it is essential to consider not only the physical problems caused by the disease but also the often-overlooked social, psychological, and behavioral challenges [18, 19]. In line with this perspective, the present study aimed to determine the relationship between fatigue experienced by patients with COPD and their psychosocial adjustment.

A review of the literature reveals a lack of studies specifically evaluating how fatigue levels in COPD patients affect their psychosocial adjustment and overall response to the disease. In this context, it is anticipated that this study will contribute significantly to the literature by supporting patients with COPD, potentially accelerating the treatment and recovery process, and enhancing patient participation and compliance.

### The research questions of the study are as follows:

1. What are the fatigue levels and psychosocial adjustment levels of patients with COPD?
2. What is the relationship between fatigue levels and psychosocial adjustment levels in patients with COPD?
3. How do fatigue levels and psychosocial adjustment levels differ according to the demographic characteristics of patients with COPD?

## Matherials and methods

### Design

This study is of descriptive type. The study was conducted in the chest diseases clinic of Cumhuriyet University Research and Application Hospital from 01.09.2023 to 01.09.2024.

### Study population and sample

The study population consisted of patients hospitalized with COPD in the chest diseases clinic at Cumhuriyet University Research and Application Hospital. The sample size was determined to be a minimum of 138 participants using the formula (*n* = t2pq/d2 (*t* = 1.96, p = 0.90, *q* = 10, *d* = 0.05), as the total population size was unknown. Between the implementation dates, 203 patients were approached. However, due to various factors such as patients being unable to speak during data collection, the presence of psychological disorders, hearing issues, or unwillingness to participate, data were ultimately collected from a total of 160 patients.

### Data collection tools

Three data collection tools were utilized in this study: a Patient Information Form developed by the researchers based on the literature, the COPD and Asthma Fatigue Scale (CAFS), and the Psychosocial Adjustment to Illness Scale-Self Report (PAIS-SR).

### Patient information form

This questionnaire, developed by the researchers based on relevant literature [10, 11, 19], includes a total of 14 questions. These questions cover age, gender, place of residence, marital status, educational status, employment status, smoking and alcohol use, cessation of work, COPD-related history, daily living activity status, social support, and treatment regimen.

### COPD and Asthma Fatigue Scale (CAFS)

The CAFS was developed by Revicki et al. [20], with its Turkish validity and reliability study conducted by Arslan and Öztunç [21]. The original scale consists of 12 items scored on a Likert-type scale (1 = never, 2 = rarely, 3 = sometimes, 4 = often, 5 = very often). The CAFS has a two-factor structure and yields a single score that represents fatigue status, without evaluating sub-dimensions. The total score ranges from 12 to 60, which can be converted to a 0–100 scale using a formula. A higher score indicates a higher level of fatigue. The Cronbach’s alpha reliability coefficient for the scale is 0.926.

### Psychosocial Adjustment to Illness Scale-Self Report (PAIS-SR)

The PAIS-SR was developed by Derogatis and Lopez in 1983 [22] to measure psychosocial adjustment to illness. It assesses how individuals interact with social and cultural institutions and with other individuals. The PAIS-SR includes 46 items across 7 subgroups: orientation to healthcare, family environment, vocational environment, extended family relationships, sexual relationships, social environment, and psychological distress. For each subgroup, four descriptive statements represent varying levels of adjustment, from which patients select the statement that best matches their experience. Items are scored on a 0–3 scale, where negative changes since illness onset receive a score of 3, and positive changes or no changes receive a score of 0. Total scores range from 0 to 138, with higher scores indicating poorer psychosocial adjustment. In studies with this scale, scores below 35 indicate good psychosocial adjustment, scores between 35 and 51 indicate moderate adjustment, and scores above 51 indicate poor adjustment. The Turkish validity and reliability study of the PAIS-SR was conducted by Adaylar [23], yielding Cronbach’s alpha values of 0.87 for Orientation to Healthcare, 0.80 for Family Environment, 0.85 for Vocational Environment, 0.89 for Extended Family Relationships, 0.95 for Sexual Relationships, 0.93 for Social Environment, 0.83 for Psychological Distress, and 0.94 for the overall scale.

### Data collection

The purpose of the study was explained to COPD patients before beginning, and their written consent was obtained. The questionnaire and scales were administered by the researchers to patients who agreed to participate, using a face-to-face interview method in the chest diseases clinic. The average administration time for the scales was 30–40 min.

### Data analysis

Data were analyzed using Statistical Package for the Social Sciences (SPSS) version 22.0. Descriptive statistics (mean, standard deviation and percentage) were used to describe demographic data and scale scores. In addition to descriptive analyses, the Independent Sample *t*-test, One-Way ANOVA, Mann–Whitney *U* test, Kruskal–Wallis test, and Spearman correlation analysis were utilized to examine differences and relationships within the data. Statistical significance was determined as *p* < 0.05.

## Results

Table 1 presents the individual characteristics of patients with COPD. The mean age of the patients was 68.70 ± 9.41 years, with 71.9% being male. Of the patients, 63.7% lived in the city center, 75.6% were married, 51.2% were primary school graduates, 75% were non-smokers, and 93.1% did not consume alcohol. Additionally, 90% of the patients were unemployed, 25% had to quit their jobs due to the disease, 67.9% had COPD for more than 6 years, and 28.7% had a family history of COPD. Furthermore, 74.4% of the patients stated that their disease interfered with daily activities, 91.9% reported having social support, and 91.2% received their treatment regularly.

**Table 1:** Distribution of ındividual characteristics of patients with COPD

Individual characteristics	Frequency	%
Average age	***68.70* ± *9.41***	
Gender		
Female	45	28.1
Male	115	71.9
Place of residence		
Urban	102	63.7
Rural	58	36.3
Marital status		
Single	39	24.4
Married	121	75.6
Educational level		
Literate	46	28.7
Primary School	82	51.2
Middle School	20	12.5
High School	8	5.0
University	4	2.5
Smoking status		
Yes	40	25
No	120	75
Alcohol consumption		
Yes	11	6.9
No	149	93.1
Current employment status		
Employed	16	10
Unemployed	144	90
Employment cessation		
Yes	40	25
No	120	75
Duration of ıllness		
1–3 years	34	21.4
4–6 years	18	10.7
7 years and above	108	67.9
Family history		
Yes	46	28.7
No	114	71.3
Impact of ıllness on daily activities		
Yes	119	74.4
No	41	25.6
Social support		
Yes	147	91.9
No	13	8.1
Adherence to regular treatment		
Yes	145	91.2
No	15	8.8
Total	**160**	**100**

Table 2 shows the distribution of mean scores for the CAFS and PAIS-SR overall and subscale scores. The mean CAFS score was 58.03 ± 15.80. The mean overall PAIS-SR score was 64.19 ± 6.41, with subscale scores as follows: orientation to healthcare (13.13 ± 2.03), vocational environment (12.19 ± 2.72), family environment (7.75 ± 2.16), sexual relationships (6.29 ± 1.74), extended family relationships (10.50 ± 1.90), social environment (4.37 ± 3.15), and psychological pressure (10 ± 2.61). The mean total scores for COPD patients on both scales were above average, indicating that patients had elevated levels of fatigue and poor psychosocial adjustment.

**Table 2:** Distribution of mean scores for CFS and PAIS-SR in patients with COPD

Scales	Mean (X)	SD	Min–max values	Possible min–max values
*CFS overall	58.03	15.80	16–98	0–100
**PAIS-SR overall	64.19	6.41	48–87	0–138
Orientation to healthcare	13.13	2.03	8–19	0–24
Vocational environment	12.19	2.72	5–19	0–24
Family environment	7.75	2.16	3–14	0–18
Sexual relationships	6.29	1.74	1–11	0–18
Extended family relationships	10.50	1.90	1–15	0–15
Social environment	4.37	3.15	0–12	0–18
Psychological distress	10.00	2.61	3–19	0–21

^*^CFS, COPD and Asthma Fatigue Scale; **PAIS-SR, Psychosocial Adjustment to Illness Scale-Self Report

Table 3 displays the correlation analysis between the CAFS and PAIS-SR mean scores. A statistically significant but weak positive correlation was found between the overall mean CAFS score, the psychological pressure subscale score, and the overall PAIS-SR mean score, suggesting that as fatigue levels increased, psychosocial adjustment worsened.

**Table 3:** Correlation of mean scores for CFS and PAIS-SR in patients with COPD

Correlation	CFS
**PAIS-SR overall**	*r* = 0.231; *p* = 0.004*
Orientation to healthcare	*r* = 0.095; *p* = 0.240
Vocational environment	*r* = 0.009; *p* = 0.907
Family environment	*r* = 0.023; *p* = 0.774
Sexual relationships	*r* = −0.142; *p* = 0.078
Extended family relationships	*r* = 0.065; *p* = 0.423
Social environment	*r* = 0.059; *p* = 0.464
Psychological distress	*r* = 0.183; *p* = 0.023*

*r* = Correlation; *Spearman correlation, *p* < 0.01

No significant correlations were found between the CAFS mean scores and the PAIS-SR subscales for orientation to healthcare, vocational environment, family environment, sexual relationships, extended family relationships, and social environment (*p* > 0.05).

Table 4 shows no significant differences (*p* > 0.05) between marital status, educational level, place of residence, disease duration, smoking status, regular treatment adherence, and the mean scale scores. However, a statistically significant difference (*p* < 0.05) was observed for gender and social support status in relation to the overall CAFS mean score, with higher scores among female participants and those lacking family support. Patients who reported that their daily activities were negatively affected by the disease had significantly higher mean total scores on both the CAFS and PAIS-SR scales (*p* < 0.05).

**Table 4:** Distribution of CFS and PAIS-SR mean scores by selected patient characteristics

Characteristic	CFS overall (X̄ ± SD)		PAIS-SR overall (X̄ ± SD)	
Gender				
Female		93.83 ± 14.71		65.51 ± 6.94
Male		55.88 ± 15.71		63.67 ± 6.15
Test statistic	Z/p	−2.741/0.006	T/p	1.63/0.708
Marital status				
Single		58.89 ± 14.73		62.87 ± 6.37
Married		57.76 ± 16.17		64.61 ± 6.39
Test statistic	Z/p	−0.128/0.898	T/p	1.48/0.139
Educational level				
Literate		60.17 ± 15.49		64.56 ± 6.28
Primary School		56.66 ± 15.52		64.53 ± 6.09
Middle School		61.56 ± 13.21		62.75 ± 5.84
High School		54.42 ± 25.91		62.87 ± 11.28
University		52.08 ± 12.84		62.75 ± 6.75
Test statistic	H/p	3.42/0.490	F/p	0.47/0.751
Place of residence				
Urban		58.35 ± 16.27		63.83 ± 6.48
Rural		57.46 ± 15.04		64.82 ± 6.29
Test statistic	Z/p	−0.565/0.572	T/p	0.94/0.347
Duration of illness				
0–3 years		54.22 ± 16.76		63.17 ± 6.81
4–6 years		59.11 ± 8.80		65.31 ± 4.93
7 years and above		58.97 ± 16.15		64.27 ± 6.31
Test statistic	H/p	0.755/0.385	F/p	3.55/0.169
Smoking status				
Yes		54.16 ± 18.72	63.20 ± 8.24	
No		59.29 ± 14.60	64.52 ± 5.67	
Test statistic	Z/p	−1.678/0.093	T/p	0.037/0.259
Impact of illness on daily life activities				
Yes		61.48 ± 14.16		64.78 ± 6.27
No		47.42 ± 16.03		62.48 ± 6.59
Test statistic	Z/p	−4.518/0.001	T/p	1.99/0.048
Social support				
Yes		57.49 ± 15.90		63.92 ± 5.95
No		63.94 ± 13.82		67.23 ± 10.13
Test statistic	Z/p	−1.299/0.194	T/p	1.79/0.075
Adherence to regular treatment				
Yes		57.84 ± 16.31		63.99 ± 6.19
No		58.77 ± 9.50		66.35 ± 8.50
Test statistic	Z/p	−0.170/0.865	T/p	1.31/0.190

*Z*, Mann-Whitney *U* test; *H*, Kruskal-Wallis test; *T*, Independent Sample *t*-test; *F*, One-way ANOVA

## Discussion

This study evaluated the relationship between fatigue levels and psychosocial adjustment in patients with COPD. To date, no other study in the literature has examined this relationship. Consequently, the study findings are discussed in light of relevant literature. It was found that patients’ fatigue levels were above average, while their psychosocial adjustment levels were low. Supporting previous studies, this study clearly shows that patients with COPD have above-average fatigue levels [11, 24] and low levels of psychosocial adjustment [25–27]. This finding suggests that COPD causes physical, emotional, and psychological challenges for patients, which, in turn, reduce psychosocial adjustment. Therefore, it is essential to plan supportive interventions, such as improving patients’ coping skills and effective use of social support systems, to address fatigue levels that may negatively impact COPD patients’ health.

Fatigue levels in COPD patients can significantly impact disease management and long-term health outcomes. Sigurgeirsdottir et al. [25] note that COPD negatively affects patients’ physical and psychosocial well-being, family relationships, and social life, underscoring the importance of healthcare professionals providing education to enhance self-management skills and develop needs-based strategies. Yang et al. [11] found that almost half of 210 COPD patients experienced moderate to severe fatigue, with patients showing more severe symptoms exhibiting worse quality of life. Similarly, studies indicate that dyspnea, depression, anxiety, and poor quality of life are key factors associated with fatigue [13, 28]. In line with the literature, this study revealed that as fatigue levels in COPD patients increased, psychosocial adjustment levels decreased. This relationship may be theoretically explained by the fact that increased fatigue depletes resources across physical, emotional, and cognitive domains, making it more difficult for patients to cope with fatigue, thereby reducing their capacity to adapt effectively to environmental and social stimuli. High fatigue levels may negatively affect psychosocial functioning by limiting patients’ participation in daily activities and social relationships. Given the lack of other studies demonstrating the relationship between fatigue levels and psychosocial adjustment, the results of this study can guide future research. This finding highlights the importance of managing fatigue symptoms and providing psychosocial support interventions for patients.

The study also observed that female patients had higher fatigue levels than males. Although some studies examining the relationship between gender and fatigue in COPD patients have reported no significant difference between groups [29–31], others have found significant differences, indicating that women with COPD may experience more severe fatigue than men [28, 32, 33]. This discrepancy may be due to biological sex differences or could be associated with an increased physical and psychosocial burden of social roles and responsibilities on women [34–37].

Studies examining fatigue levels in COPD patients have identified social support as an important determinant among sociodemographic variables[6, 38, 39]. Hattab et al. [40] listed poor social support at home as a major factor contributing to hospitalizations. Similarly, Sigurgeirsdottir et al. [25] interpreted decreased participation in family and social life as the loss of ability to engage in ordinary and meaningful activities. Turnier et al.[41] also showed that increased social support is significantly associated with a lower probability of depressive symptoms, which, in turn, is linked to better COPD outcomes. Barton et al. [42] noted that adequate perceived social support has a positive effect on mental health and self-efficacy in COPD patients. These findings support the results of our study, which found that patients lacking family support had higher levels of fatigue. Family support can reduce stress by providing both emotional and physical assistance during the chronic disease process, facilitating psychosocial adjustment to treatment. The absence of social support likely intensifies feelings of fatigue by increasing the risk of loneliness, social isolation, and psychological distress.

Another factor related to fatigue and psychosocial adjustment in COPD patients, as documented in the literature, is the negative impact on daily living activities. Studies report that patients with high fatigue levels experience limitations in performing daily living activities due to reduced physical functionality [6, 30, 43]. Paddison et al. [30] found that patients with higher fatigue levels had more limitations in their activities of daily living (ADLs). Tödt et al. [37] demonstrated that exercise capacity and disease severity in COPD patients were associated with fatigue in both men and women. Michalovic et al. [44] observed that, although COPD patients wished to increase participation in daily and social activities, they were often restricted due to shortness of breath. Therefore, strategies to alleviate shortness of breath should be identified and incorporated into patients’ daily routines to help them achieve their participation goals in daily and social activities. In our study, we observed that patients with high fatigue levels experienced greater limitations in their daily activities. This finding reflects the negative impact of fatigue on physical functioning, as fatigue can limit energy levels and general mobility, making it difficult for patients to maintain daily activities. This suggests that fatigue in chronic conditions like COPD is not merely a symptom but a significant factor that profoundly affects patients’ quality of life.

All findings from our study reveal a direct relationship between fatigue levels and psychosocial adjustment in COPD patients. Additionally, gender, social support status, and daily living activity levels were found to be associated with both fatigue and psychosocial adjustment. These findings highlight the need for further research to better understand the relationship between fatigue and psychosocial adjustment and to explore the implications of these results.

### Limitations

This study has several limitations. The sample size and research area were restricted, as the study was conducted in a single center. Additionally, some participants who initially agreed to take part later withdrew due to shortness of breath during the survey, which impacted the study’s applicability.

## Conclusion

In conclusion, it was found that COPD patients experience moderate levels of fatigue and low psychosocial adjustment. A significant positive relationship was observed between fatigue levels and psychosocial adjustment in COPD patients, indicating that as fatigue levels increase, psychosocial adjustment to the disease declines. To improve the quality of care for COPD patients and alleviate fatigue levels, interventions that involve both the patient and their social environment should be implemented to enhance psychosocial adjustment. Future studies should consider a larger sample size and broader research scope to validate and expand these findings.

## Data Availability

The data supporting the findings of this study are available from the corresponding author upon reasonable request.
